# Supplementary material for: The E3 Ubiquitin Ligase NEDD4-1 Mediates Temozolomide-Resistant Glioblastoma through PTEN Attenuation and Redox Imbalance in Nrf2–HO-1 Axis
Source: Int J Mol Sci. 2021 Sep 23;22(19):10247. doi: 10.3390/ijms221910247 (PMC8549703; doi:10.3390/ijms221910247)
Supplement: Supplementary file 1 [file ijms-22-10247-s001.zip › ijms-1339215-supplementary.pdf]

Supplementary Table S1. Western blot antibodies sheet.

No.	Target	Dilution	MW	Source	
1	NEDD4-1	1:1000	115	#2740	cellsignaling
2	PTEN	1:1000	54	#9188	cellsignaling
3	p-Akt	1:1000	60	#4060	cellsignaling
4	Akt	1:1000	60	#9272	cellsignaling
5	SOX2	1:1000	40	ab97959	abcam
6	NESTIN	1:1000	177	ab105389	abcam
7	N-Cadherin	1:1000	140	#13116	cellsignaling
8	E-Cadherin	1:1000	135	#3195	cellsignaling
9	Vimentin	1:1000	57	#5741	cellsignaling
10	Hrf2	1:1000	23	sc-133131	SANTA CRUZ
11	HO-1	1:1000	32	sc-390991	SANTA CRUZ
12	Ki-67	1:500	358	ab16667	abcam
13	β-actin	1:10000	42	66009-1-Ig	PROTEINTECH

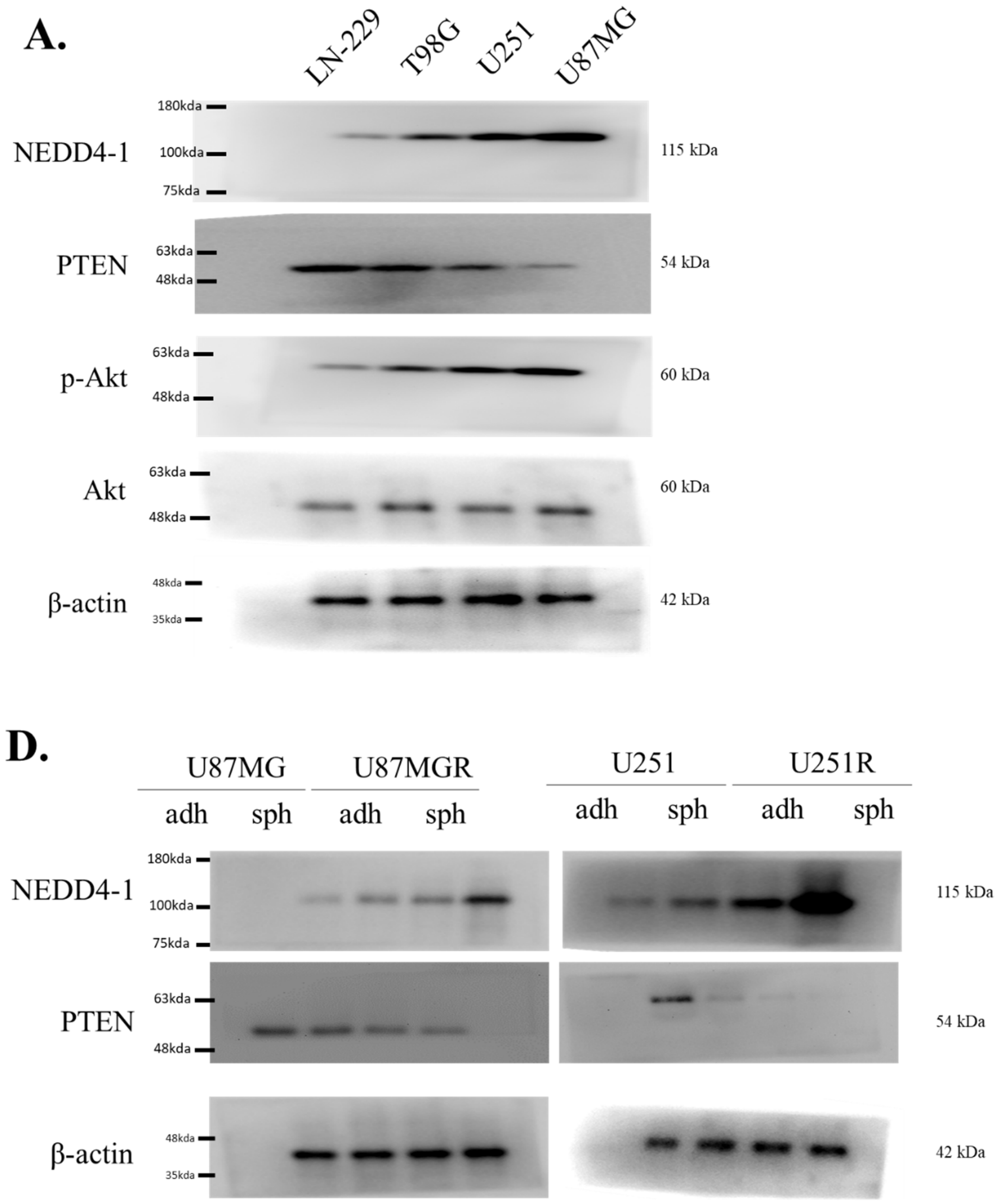

Supplementary Figure S1. Full-size blots of Figure 2A and 2D

E.

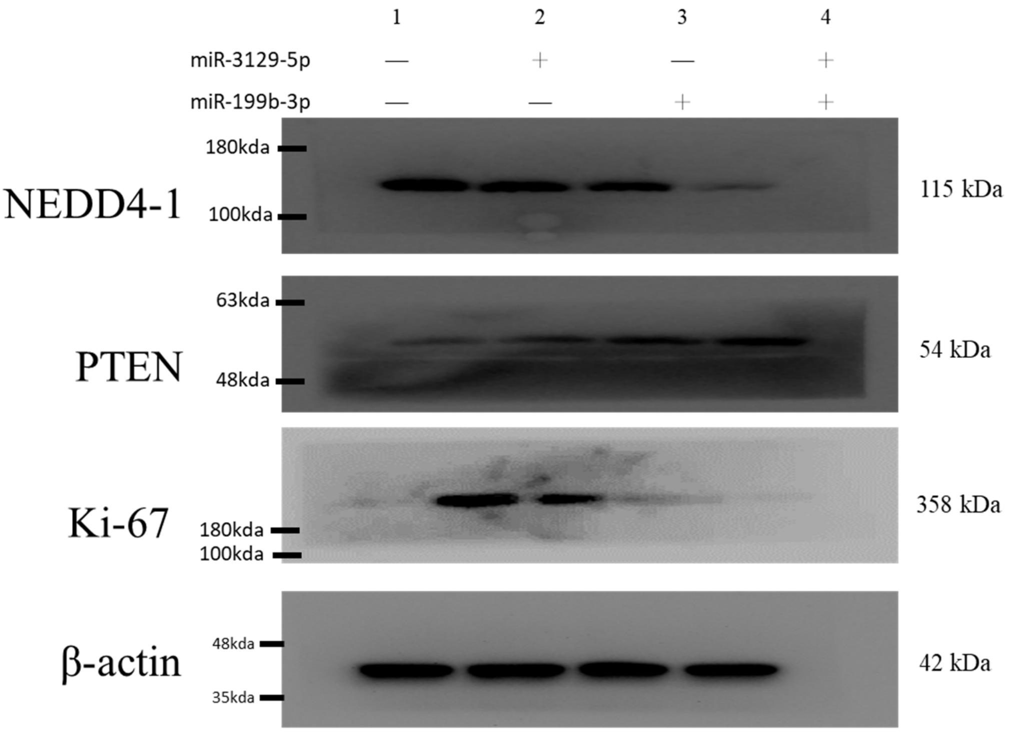

Supplementary Figure S2. Full-size blots of Figure 3E

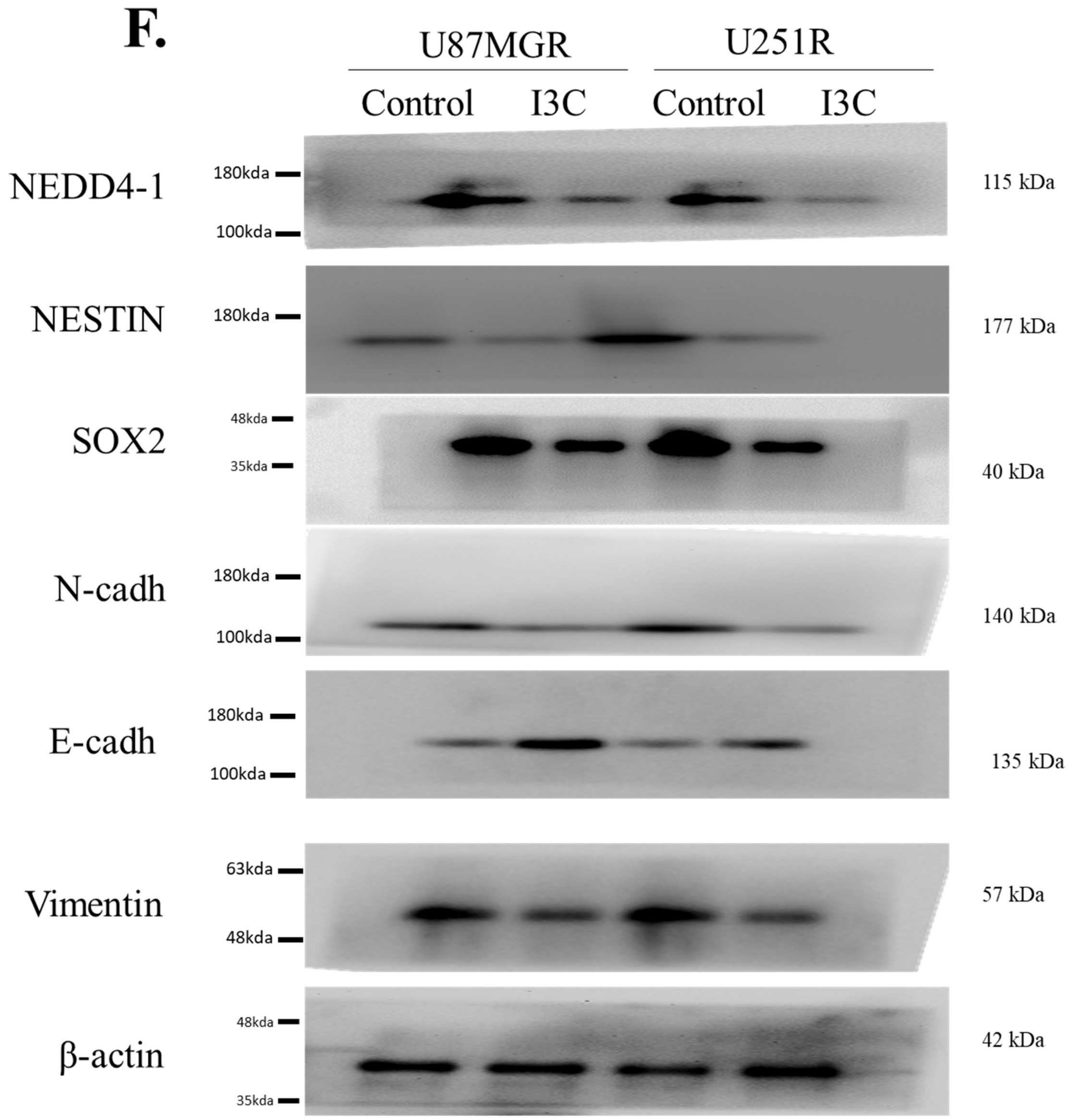

Supplementary Figure S3. Full-size blots of Figure 4F

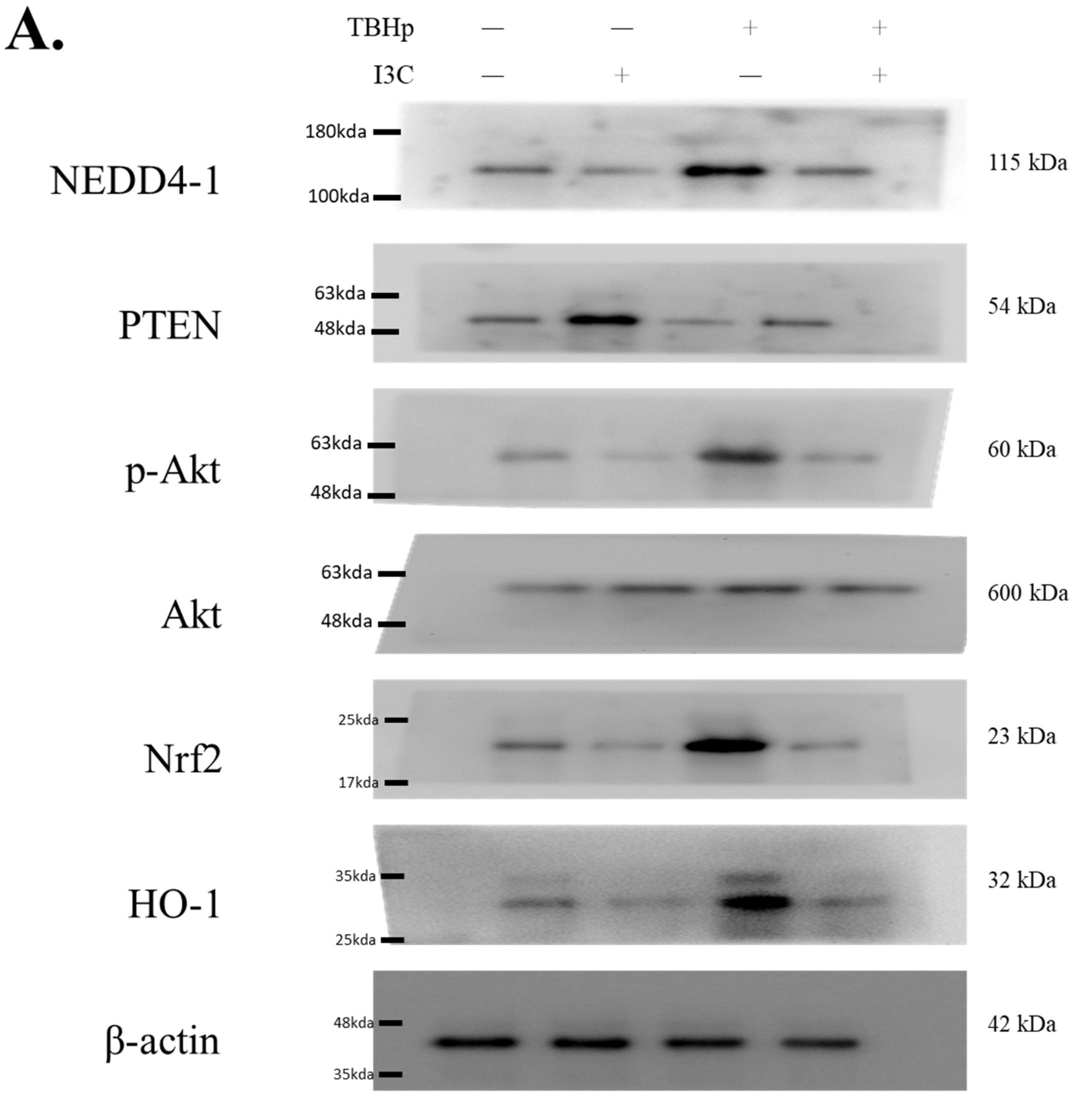

Supplementary Figure S4. Full-size blots of Figure 5A
